# Therapeutic drug monitoring in children and adolescents with schizophrenia-spectrum, affective, behavioural, tic and other psychiatric disorders treated with aripiprazole: results of the TDM-VIGIL pharmacovigilance study

**DOI:** 10.1007/s00702-024-02819-6

**Published:** 2024-11-02

**Authors:** Jessica Riegger, Karin Maria Egberts, Hans-Willi Clement, Katja Schneider-Momm, Regina Taurines, Stefanie Fekete, Christoph Wewetzer, Andreas Karwautz, Christoph U. Correll, Paul L. Plener, Uwe Malzahn, Peter Heuschmann, Stefan Unterecker, Maike Scherf-Clavel, Hans Rock, Gisela Antony, Wolfgang Briegel, Tobias Banaschewski, Tobias Hellenschmidt, Michael Kaess, Michael Kölch, Tobias Renner, Christian Rexroth, Gerd Schulte-Körne, Susanne Walitza, Manfred Gerlach, Marcel Romanos, Christian Fleischhaker

**Affiliations:** 1 Department of Child and Adolescent Psychiatry and Psychotherapy, University Medical Center Freiburg, Freiburg, Germany; 2 Department of Child and Adolescent Psychiatry, Psychosomatics and Psychotherapy, Center for Mental Health, University Hospital of Wuerzburg, Wuerzburg, Germany; 3 Clinic for Child and Adolescent Psychiatry Holweide, Kliniken der Stadt Köln GmbH, Children’s Hospital Amsterdamer Straße, Cologne, Germany; 4 Present Address: KIRINUS Tagesklinik Nymphenburg, Munic, Germany; 5 Department of Child and Adolescent Psychiatry, Medical University Vienna, Vienna, Austria; 6 Department of Child and Adolescent Psychiatry, Charité Universitätsmedizin Berlin, Berlin, Germany; 7 Department of Psychiatry, The Zucker Hillside Hospital, Northwell Health, Glen Oaks, NY USA; 8 Department of Psychiatry and Molecular Medicine, Donald and Barbara Zucker School of Medicine at Hofstra/Northwell, Hempstead, NY USA; 9 Department of Child and Adolescent Psychiatry/Psychotherapy, University Hospital Ulm, Ulm, Germany; 10 Clinical Trial Center Wuerzburg, University Hospital Wuerzburg, Wuerzburg, Germany; 11 Institute of Clinical Epidemiology and Biometry, University of Wuerzburg, Wuerzburg, Germany; 12 Department of Psychiatry, Psychosomatics and Psychotherapy, Center of Mental Health, University Hospital Wuerzburg, Wuerzburg, Germany; 13 Central Information Office, Department of Neurology, Philipps University of Marburg, Marburg, Germany; 14 Department of Child and Adolescent Psychiatry, Psychosomatics and Psychotherapy, Leopoldina Hospital, Schweinfurt, Germany; 15 Department of Child and Adolescent Psychiatry and Psychotherapy, Medical Faculty Mannheim, Central Institute of Mental Health, Heidelberg University, Mannheim, Germany; 16 Department of Child and Adolescent Psychiatry, Psychotherapy and Psychosomatic medicine, Vivantes Clinic Berlin Neukölln, Berlin, Germany; 17 Clinic for Child and Adolescent Psychiatry, Center for Psychosocial Medicine, University Hospital Heidelberg, Heidelberg, Germany; 18 University Hospital of Child and Adolescent Psychiatry and Psychotherapy, University of Bern, Bern, Switzerland; 19 Department of Child and Adolescent Psychiatry and Psychotherapy, Brandenburg Medical School Brandenburg, Neuruppin, Germany; 20 Department of Child and Adolescent Psychiatry, Neurology, Psychosomatics, and Psychotherapy, University Medical Center Rostock, Rostock, Germany; 21 Department of Child and Adolescent Psychiatry, Psychosomatics and Psychotherapy, University Hospital of Psychiatry and Psychotherapy Tuebingen, Center of Mental Health Tuebingen, Tuebingen, Germany; 22 Clinic for Child and Adolescent Psychiatry, Psychosomatics and Psychotherapy, University of Regensburg at the Regensburg District Hospital, Medbo, Regensburg, KU Germany; 23 Department of Child and Adolescent Psychiatry, Psychosomatics and Psychotherapy, Ludwig-Maximilians-University (LMU) Hospital, Munich, Germany; 24 Department of Child and Adolescent Psychiatry and Psychotherapy, Psychiatric University Hospital Zurich, University of Zurich, Zürich, Switzerland; 25 Zurich Center for Integrative Human Physiology, University of Zurich, Zürich, Switzerland; 26 Neuroscience Center Zurich, University of Zurich and ETH, Zürich, Switzerland

**Keywords:** Aripiprazole, Children and adolescents, Serum concentration, Schizophrenia, Affective disorder, Therapeutic drug monitoring

## Abstract

Aripiprazole is approved for various severe mental disorders in adults and adolescents. However, off-label prescribing is common, especially in children and adolescents (youth) in whom aripiprazole therapeutic serum level reference ranges are lacking for any disorders. The aim of the study was to evaluate the relationship between aripiprazole dose and serum concentrations and provide further knowledge on the use of aripiprazole in order to improve drug safety and effectiveness in the treatment of minors. The clinical course of youth treated with aripiprazole in the multicentre pharmacovigilance study TDM-VIGIL was systematically followed and serum concentrations measured. Sex, age, weight and comedications were analysed to identify possible effect modifiers. A preliminary therapeutic reference range was estimated for youth with schizophrenia-spectrum disorders, affective disorders and behavioural/emotional/tic disorders coded as treatment responders based on a Clinical-Global Impressions-Improvement (CGI-I) score of much or very much improved. In 93 youth (mean age = 15.2 ± 2.6, range = 7.4–18.2 years, females = 53%, CGI-Severity = 4.4 ± 1.1, responders = 64%), a positive, moderate correlation between the weight-normalized daily dose (WNDD) and aripiprazole serum concentration (=0.791, *p* < 0.0001) was found. The WNDD and co-medications that interact with CYP2D6 and CYP3A4 affected aripiprazole serum levels, explaining 64% of the variance. In patients within the preliminary therapeutic ranges determined by interquartile ranges (IQRs), slightly better outcomes and fewer adverse drug reactions were found versus patients within preliminary therapeutic ranges determined by the mean ± SD. The preliminary reference range for paediatric patients with schizophrenia-spectrum disorders calculated by the IQR showed an identical lower threshold (100–230 ng/ml) compared to adult schizophrenia-spectrum disorders patients (100–350 ng/ml). The preliminary therapeutic ranges for patients with affective disorders was: 60–160 ng/ml and for patients with behavioural/tic disorders 60–140 ng/ml. The therapeutic reference ranges for aripiprazole in youth estimated via the 25th and 75th IQRs may result in more clinically relevant therapeutic windows. Further studies need to confirm these results, especially in patients with affective and behavioural/tic disorder diagnoses.

## Introduction

Aripiprazole, a partial D_2_- and 5-HT_1A_-receptor agonist and 5-HT_2A_ antagonist (Jordan et al. 2002; Kane et al. 2002), has regulatory approval for treating adults and adolescents aged 13–17 (USA) and 15–17 (Europe) years with schizophrenia as well as bipolar I disorder and manic or mixed episodes in adults and youth aged 10–17 years (USA) (Otsuka Pharmaceutical 2020). For patients aged 13–17 with schizophrenia or with bipolar I disorder aged 10–17 years, the recommended dose is 10 mg/d, and the maximum dose of 30 mg/d should not be exceeded (Otsuka Pharmaceutical 2020). In addition to the acute efficacy, long-term treatment efficacy for maintenance and relapse prevention has been established with aripiprazole both schizophrenia in paediatric patients aged 13–17 years old (Correll et al. 2017) and with bipolar I disorder aged 10–17 years old (Findling et al. 2013). For manic episodes in the context of a bipolar I disorder, studies have suggested 15 mg/d up to 30 mg/d to be efficacious in the treatment of adults (Keck et al. 2003; Sachs et al. 2006). In the US, aripiprazole is also approved to treat paediatric patients starting at age 7 with Tourette’s disorder, and starting at age 6 for irritability associated with autism-spectrum disorders. Although aripiprazole is not approved by the European Medicines Agency for these two disorders in children and adolescents, it is widely used for these indications in European countries (Pozzi et al. 2016), Hirsch and Pringsheim 2016). There is also a common off-label use of aripiprazole in psychiatric patients of all ages with various ailments, including disruptive disorders with increased impulsive behavioural phenotype, personality disorders, or adjunctive treatment for major depressive disorder. In this context, ongoing research is investigating the safety and efficacy of aripiprazole in patients with depression (Lai et al. 2016; Marcus et al. 2008; Ozaki et al. 2015).

The treatment of children and adolescents with aripiprazole comes with several challenges. First, it has been found that adverse drug reactions (ADRs) of antipsychotics, such as weight gain, as well as drowsiness and extrapyramidal side effects (EPMS) pose a higher risk in the treatment of children and adolescents with antipsychotics compared to adults (Koch et al. 2023; Kryzhanovskaya et al. 2012; Correll and Carlson 2006; Pringsheim et al. 2011; Al-Dhaher et al. 2016), although some of the risk may be attenuated with careful titration and dosing of second-generation antipsychotics (Carbon et al. 2015). Second, the off-label treatment of illnesses, for which paediatric doses have not been established yet, exposes children and adolescents to the risk of dosing errors (Conn et al. 2019), as explorations of medication effects in minors are often inadequate (Schröder et al. 2017). Systematic clinical studies in children and adolescents are both time-consuming and expensive due to ethical and practical requirements, and are consequently available for only few substances (Egberts et al. 2015). The treatment of children and adolescents with psychotropic drugs must be differentiated from that of adults though, as their ongoing growth causes differences in the physiology and metabolism as well as pharmacodynamics and pharmacokinetics of medications (Gerlach et al. 2014). Third, due to the metabolism of aripiprazole by the liver via the hepatic enzymes CYP2D6 (predominantly) and CYP3A4 (Wood et al. 2006), pharmacokinetic interactions with this enzyme system may occur, if inhibiting or inducing comedications are administered (Spina and De Leon 2007; Conley and Kelly 2007; Kapfhammer et al. 2010). Although there are only few studies on polypharmacy in children and adolescents, they indicate a relatively high prevalence of polypharmacy in patients with schizophrenia-spectrum disorders (Toteja et al. 2014; Constantine et al. 2010). Percentages of up to 65% and 97% were reported in different studies, depending on the main indication and definition of polypharmacy (Bachmann et al. 2008; Vloet et al. 2019). In addition, polymorphisms may influence the regular metabolism of aripiprazole and its clearance significantly, which can result in as much as twice as long elimination half-lives in so-called “poor metabolizers” (Kim et al. 2008; Kapfhammer et al. 2010). In order to minimize these potential risks, besides a strict indication, pharmacovigilance measures are helpful, which are intended to contribute to the effectiveness of therapy by detecting and preventing ADRs and other drug-related safety issues (Egberts et al. 2015; Gerlach et al. 2016; Schoretsanitis et al. 2020).

Therapeutic drug monitoring (TDM) describes the practice of dosing a medication while regularly measuring serum concentration of the drug and observing the clinical effects (Schoretsanitis et al. 2020; Hiemke et al. 2018). In this manner, certain blood concentrations, the so called “therapeutic reference ranges”, are targeted, at which a positive clinical effect is to be expected while the risk of ADRs is kept to a minimum (Baumann et al. 2004; Hiemke et al. 2011, 2018; Egberts et al. 2015). TDM has been found to be an effective tool for the safety of psychotropic drugs in children and adolescents and has been suggested as a potentially helpful tool in addition to routine care when administering psychotropic drugs to minors (Hiemke et al. 2018; Egberts et al. 2021). It is also suggested, that by verifying serum levels within the reference range and thereby improving the likelihood of the therapeutic benefit, TDM has the potential to reduce non-adherence and the recurrence of a psychiatric episodes (Stieffenhofer et al. 2011; Hiemke et al. 2018) as well as the occurrence of EPMS, as has been shown for other substances in children and adolescents, e.g. risperidone (Taurines et al. 2022; Kloosterboer et al. 2021).

In adult psychiatry, various psychotropic drugs have already been well studied, and therapeutic reference ranges have been established (Hiemke et al. 2018). In child and adolescent psychiatry, therapeutic reference ranges are still scarce. In the recent past, first attempts to determine therapeutic reference ranges for aripiprazole in children and adolescents with schizophrenia-spectrum disorders have been proposed. However, no definitive reference range has been established or accepted so far, nor have other diagnoses been examined further with TDM. Moreover, no common rule of how to calculate the lower and upper limit of such therapeutic serum concentration ranges has been acknowledged. The recommended reference range for aripiprazole for adults with schizophrenia (100–350 ng/ml) has also not yet been confirmed for children and adolescents.

Therefore, the aim of this study was to contribute to the safety, efficacy and effectiveness of aripiprazole in children and adolescents by further investigating the relationship between the serum concentrations and doses, focusing on finding characteristics, such as age, sex or comedications, which may lead to different pharmacokinetic outcomes. In addition, this study sought to define therapeutic reference ranges for paediatric patients with different diagnoses in order to determine whether or not there might be reasons to treat patients with distinct disorders with different dose ranges.

## Methods

### Study design and study population

Patient and medication data were derived from the multicentre pharmacovigilance trial (Phase IIIb) ‘TDM-VIGIL’, subproject 1, EudraCT number 2013-004881-33, which prospectively collected data from 710 children and adolescents with different psychiatric and behavioural disorders treated naturalistically between May 2014 and December 2018, described in detail elsewhere (Egberts et al. 2022). The samples were analysed at the specialized TDM laboratory of the centre of mental health of the university hospital Wuerzburg according to standardized and validated methods using HPLC separation and UV detection. Patient information, such as demographic, psychiatric and clinical outcome data as well as medication regimens, were collected in an online database. Inclusion criteria were limited to psychiatric patients aged 4 to 18 years from an outpatient, day-hospital and inpatient treatment settings who initiated antidepressants and antipsychotics in on-label or off-label use and with a valid written ‘informed consent’. Patients with an existing absolute contraindication for the administration of the medication, and those participating in another clinical trial were excluded. The study was approved by the ethics committees of all participating centres (lead study centre University Hospital Wuerzburg; 301/13).

### Data collection

Upon admission to the study, basic information such as sex, age, clinical psychiatric diagnoses, comorbidity (according to the German Modification of the International Classification of Diseases) and nicotine use were recorded. Data concerning physical-neurological and psychiatric examination, vital signs, body weight and height, as well as laboratory analyses for hepatic and renal function were also collected at baseline and at every clinically driven blood withdrawal visit. In addition, clinical data of the medication treatment (e.g. dosage of the drug, type and dosage of any psychiatric co-medications) were documented. As for the TDM analysis, date, indication and symptoms intended to treat with the medication were documented.

For the aripiprazole study sample, in addition to the general inclusion/exclusion criteria predetermined by the TDM-VIGIL trial (see methods and materials), further exclusion criteria were applied. Several patients were examined during TDM more than once during the course of treatment. In these cases, only the last available valid measurement was included in the analysis. If the online register documented errors in samples, these were also discarded from the aripiprazole study sample. Such errors could include: intoxication, administration errors, lack of plausibility, uncertain adherence, or other errors during blood sampling. To avoid bias, patients were excluded if their available data interfered with quality criteria for the study (http://www.prisma-statement.org). Additionally, the following quality criteria for TDM-studies were adapted (Ulrich et al. 1998; Kloosterboer et al. 2020): standardized analytical method (described below), steady state conditions during blood withdrawal, representative sample selection of patients, quantification of the illness severity, and sample sizes of at least ten or more individuals (Ulrich et al. 1998; Kloosterboer et al. 2020). It also was required that enough time had passed between baseline and the next visit with measurement of the change in the clinical condition to be able to adequately assess the effect of the medication. Patients were furthermore screened for the use of any CYP2D6 and CYP3A4 inhibitors in order to allow comparisons between patients with and without such inhibitors (Hiemke et al. 2018).

Clinical effectiveness was measured by the Clinical Global Impression Scale (CGI) (Guy 1976), evaluating the severity of psychopathology (CGI-S) at baseline/before medication and the change since the baseline visit (CGI-I) at the day of blood sampling. Categories of this latter scale range from: 1 = very much improved, 2 = much improved, 3 = minimally improved, 4 = unchanged, 5 = minimally worse, 6 = much worse and 7 = very much worse. Patients with a CGI-I score of 1 and 2 were defined as treatment responders. In addition to the CGI, the clinical efficacy (NET-CGI) of the medication was determined, ranging from: 1 = very good (improvement is extensive, symptoms are (almost) completely remitted), 2 = moderate (improvement is clear, symptoms are partially remitted), 3 = slight (improvement is mild, patient needs further treatment), and 4 = no improvement of (or rather burdened by) the condition). To collect data on ADRs, the Pediatric Adverse Events Rating Scale (PAERS) (March et al. 2007) was used, using the characterization 1 = mild, 2 = moderate, 3 = severe, 4 = extremely severe. ADRs were defined as adverse events with at least a possible association with the study drug reported by the treating physician.

### Determination of preliminary therapeutic reference ranges

As there are yet no validated therapeutic reference ranges for psychotropic drugs for children and adolescents, the consensus guidelines on therapeutic drug monitoring (Hiemke et al. 2011) proposed how to determine preliminary therapeutic reference ranges until fixed reference ranges are validated. According to this method, the mean value ± the standard deviation (SD) of the serum concentrations of responding patients, in our study subjects with CGI-I scores of 1 and 2, is calculated as the preliminary therapeutic range. This should only be done when data are normally distributed. Another commonly used method defines the therapeutic reference range via the 25th and 75th interquartile range (IQR) of the median of serum concentrations measured in patients categorized as treatment responders. This method should be used when data are not normally distributed and was for example utilized by Kirschbaum et al. (2008) in order to determine the valid therapeutic reference range of aripiprazole in adults (100–350 ng/ml). It has also been suggested to exclude responders who have been treated with relevant cytochrome P450 enzyme inhibitors to avoid interactions with aripiprazole metabolism (Hart et al. 2021; Ulrich et al. 1998; Kloosterboer et al. 2020).

The preliminary therapeutic reference ranges in our study were estimated from serum concentrations of treatment responders without CYP2D6- and CYP3A4-inhibiting comedications by the mean ± SD, as suggested by the AGNP Consensus Guidelines (Hiemke et al. 2018), and also by the 25th and 75th IQR, as practiced by other authors before (Kirschbaum et al. 2008; Bachmann et al. 2008) and recommended by the recent review of Hart and colleagues (2021).

### Data analysis

Statistical analysis and graphs were completed with R® for Windows (Version 4.0.3., RStudio PBC 250 Northern Ave, Boston, MA 02210) and Microsoft Excel® (Version 15, Microsoft, Microsoft Deutschland GmbH, Munich). All values are presented as mean ± SD or median [IQR]. Testing for Gaussian distribution was performed using the Shapiro-Wilk test and graphical analysis to check the result of the test. Pearson’s coefficient (*r*) for normally distributed and Spearman’s rank correlation coefficient (*r*_*s*_) for not normally distributed values were used to compute correlations between aripiprazole dosage and serum concentrations and other values. Differences between two Gaussian distributed subgroups were tested using the t-test for independent samples with equal variances, or with the Welch test for independent samples with unequal variances. Variances were explored via the Levene test. The Wilcoxon-Mann-Whitney U test was used for not normally distributed variables. In addition, the Kruskal-Wallis test, was used to compute differences between more than two subgroups. Chi2 tests were used for analysis of categorical variables, using Fisher’s test when any cell had ≤ 5 cases. Statistical significance was defined as *p* < 0.05 and adjusted for multiple testing according to Bonferroni’s method. Subgroup-stratified analyses were conducted when >/=10 patients were in the relevant subgroup.

Multiple linear regression models were fitted to evaluate the relationship between aripiprazole serum concentration as the target variable and weight-adjusted aripiprazole dose as the predictor, adjusting for age, sex and weight. These parameters were selected in order to be able to make clinical recommendations in case of any significant differences.

## Results

### Study population

Of the original 133 patients, 37 were excluded from the analysis because their blood samples were not collected during steady state conditions. Three additional patients were excluded because their data did not meet the PRISMA criteria: One patient because the time interval between baseline and the following assessment was not plausible, and two patients because disease severity assessment had not been performed and documented at baseline. Consequently, the aripiprazole study population included a total of 93 patients (47% male) with a mean age of 15.2 ± 2.6 (range 7.4–18.2) years, with the vast majority being older than 13 years (74%).

Patients were treated with aripiprazole for different psychiatric diagnoses, including schizophrenia-spectrum disorders, accounting for the largest proportion (33%, mean age 16.2 ± 1.3 (range: 13.7–18.) years, 55% males), followed by affective disorders (28%, mean age 16.0 ± 1.5 (range: 12.2–18.2) years, 15% males), and behavioural, emotional or tic disorders (24%, mean age 12.3 ± 2.9 (range 7.4–17.6) years, 68% males). Additional diagnoses included anxiety disorders (15%), personality disorders /traits (12%) and “other” diagnoses (22%), with some being comorbidities. All diagnoses are listed in Table 1 along with the main characteristics of the study population. Patients were on average moderately ill (CGI-S: 4.4 ± 1.1) and mostly treated as inpatients (83%) or in the day hospital (13%), with fewer outpatients (4%).

**Table 1:** Characteristics of the study population (*n* = 93)

Characteristic	*N* (%) or mean ± standard deviation
**Clinical centre**, *N* (*%*)	
Berlin Charité University Hospital	4 (4.3)
Berlin Vivantes	1 (1.1)
Freiburg University Hospital	8 (8.6)
Heidelberg University Hospital	5 (5.4)
Cologne	1 (1.1)
Mannheim ZI University Hospital	5 (5.4)
Munich LMU University Hospital	1 (1.1)
Neuruppin, Ruppiner Kliniken	5 (5.4)
Regensburg Bezirksklinikum	2 (2.2)
Schweinfurt Leopoldina Hospital	8 (8.6)
Tuebingen University Hospital	3 (3.2)
Ulm University Hospital	11 (11.8)
Vienna University Hospital	4 (4.3)
Wuerzburg University Hospital	27 (29.0)
Zuerich University Hospital	8 (8.6)
**Sex**, *N* (*%*)	
Male	44 (47.3)
Female	49 (52.7)
**Age** (years), mean ± SD, range	15.2 ± 2.6, 7.4–18.2
Children < 14 years, *N* (*%*)	24 (25.8)
Adolescents ≥ 14 years, *N* (*%*)	69 (74.4)
**Weight** (kg), *N* = 93, mean ± SD, range	61.8 ± 18.0, 24.7–112.0
**Height** (cm), *N* = 93, mean ± SD, range	164.3 ± 13.3, 126.0–185.0
**BMI** (kg/m2), *N* = 93, mean ± SD, range	22.5 ± 4.9, 15.0–38.8
**BMI Percentiles P**, *N (%)*	
Underweight (< P10)	5.4
Normal weight (P10-90)	66.7
Overweight (> P90-97)	9.7
Obese (> P97-99,5)	12.9
Extreme obese (> P99.5)	5.4
**Smoking**, *N* (*%*)	13 (14.0)
**Treatment Setting**, *N* (*%*)	
Inpatient	82.8
Day hospital	12.9
Outpatient	4.3
**Diagnoses/Comorbidities**, *N* (*%*)	
Schizophrenia, schizotypal, delusional, and other non-mood psychotic disorders (F 20–29)	31 (33.3)
Affective disorders (F 30–39)	26 (28.0)
Behavioural, emotional and tic disorders (F 90–99)	22 (23.7)
Behavioural disorders (F90-92)	15
Unspecified disorder of social functions (F94)	3
Emotional disorders with onset specific to childhood (F93)	2
Tic disorders (F95)	2
Anxiety disorders (F 40–48)	14 (15.1)
Personality disorders (F 60–69)	11 (11.8)
**Other**	
Unspecified organic or symptomatic mental disorder (F09)	1 (1.1)
Mental and behavioural disorders due to psychoactive substance use (F10–F19)	3 (3.2)
Behavioural syndromes associated with physiological disturbances and physical factors (F50–F59)	5 (5.4)
Mental retardation (F70–F79)	3 (3.2)
Behavioural and emotional disorders with onset usually occurring in childhood and adolescence (F80–F89)	8 (8.6)
**Severity of illness (CGI-S)**, *N* = 93, mean ± SD	4.4 ± 1.1
Not assessable, *N* (*%*)	/
Not at all ill, *N* (*%*)	/
Borderline mentally ill, *N* (*%*)	5 (5.4)
Mildly ill, *N* (*%*)	10 (10.8)
Moderately ill, *N* (*%*)	33 (35.5)
Markedly ill, *N* (*%*)	33 (25.5)
Severely ill, *N* (*%*)	10 (10.8)
Extremely ill, *N* (*%*)	2 (2.2)
**Improvement (CGI-I)**, *N* = 93, mean ± SD	2.3 ± 1.1
Not assessed/assessable, *N* (*%*)	1 (1.1)
Very much improved, *N* (*%*)	17 (18.3)
Much improved, *N* (*%*)	41 (44.1)
Minimally improved, *N* (*%*)	23 (24.7)
Unchanged, *N* (*%*)	7 (7.5)
Minimally worse, *N* (*%*)	3 (3.2)
Much worse, *N* (*%*)	1 (1.1)
Very much worse, *N* (*%*)	/
**Clinical efficacy of the test medication**, *N* = 91	
Not assessed/assessable, *N* (*%*)	2 (2.2)
very good, ‘improvement is extensive, symptoms are (almost) completely remitted’	21 (22.6)
moderate, ‘improvement is clear, symptoms are partially remitted’	39 (41.9)
slight, ‘improvement is easy, patient needs further treatment’	23 (24.7)
no improvement, ‘(or rather burdened) of the condition’	8 (8.6)
**Aripiprazole monotherapy**, *N* (*%*)	25 (26.9)
**Psychiatric co-medications**, *N* (*%*)	
One co-medication	40 (43.0)
More than one co-medication	28 (30.1)
**Psychiatric co-medication**, *N* (*%*), multiple entries	
Antipsychotics (chlorprothixene, clozapine, haloperidol, olanzapine, pipamperone, promethazine, quetiapine, prolonged-release quetiapine, risperidone, zuclopenthixol)	23 (24.7)
Antidepressants (citalopram, escitalopram, fluvoxamine, fluoxetine, mirtazapine, paliperidone, sertraline, prolonged-release venlafaxine)	34 (36.6)
Mood stabilizer, anticonvulsants (lamotrigine, prolonged-release valproate)	2 (2.2)
Tranquilizer (diazepam, lorazepam, valerian root)	2 (2.2)
Stimulants (amphetamine, lisdexamfetamine, methylphenidate, extended-release methylphenidate)	12 (12.9)
Biperiden	3 (3.2)
Other	12 (12.9)
Cytochrome P CYP2D6 Inhibitor (fluoxetine, promethazine & sertraline)	20 (21.5)
Cytochrome P CYP3A4 Inhibitor (fluvoxamine)	1 (1.1)
**Non-Psychiatric co-medications**, *N* (*%*), multiple entries	16 (17.2)

Altogether, 74% of patients continued their aripiprazole administration after the end of the study, while only about a quarter of all patients discontinued the medication during and after the study. Almost two-thirds of the patients (58%) received concomitant psychotropic medications. The frequencies of polypharmacy did not differ significantly with regard to sex (girls 78%, boys 68%), age (children 71%, adolescent 74%) or diagnosis (schizophrenia-spectrum disorders 68%, affective disorders 88%, personality disorders 82%, behavioural, emotional or tic disorders 64%). Less than a quarter of patients (23%) were concomitantly treated with the CYP2D6 inhibitors fluoxetine, promethazine or sertraline, or the CYP3A4 inhibitor fluvoxamine, which carry the risk of clinically relevant drug-drug interactions with aripiprazole. The frequency of inhibiting comedications between girls and boys (girls 25%, boys 2%) or children and adolescents (children 17%, adolescent 25%), did not differ widely, but there was a more noticeable difference between the schizophrenia-spectrum disorders (3%), affective disorders (27%) and personality disorders (18%). No patient in our sample received a cytochrome P450 enzyme-inducing comedication.

### Aripiprazole serum concentrations in relation to daily dose and weight-adjusted daily dose

Aripiprazole was prescribed in flexible daily doses between 2.0 and 30.0 mg/d with an average of 9.5 mg/d (± 6.0). Dosages (mean ± SD; median; IQR) for the total sample and the various subsamples can be found in Table 2. We observed no significant difference between daily doses of girls and boys or monotherapy and polypharmacy. However, patients under the age of 14 were treated with lower aripiprazole doses than those aged 14 and above (mean 7.3 mg vs. 10.2 mg; *p* = 0.01).

**Table 2:** Daily doses (in mg/day), weight-adjusted doses (in mg/kg) and serum concentrations (in ng/ml) of aripiprazole in different subsamples, including correlation coefficients and level of significance of values between subgroups

Patients (*N*)	Daily dose mean ± SD, median, (IQR) (mg/day)	Weight-adjusted daily dose mean ± SD, median, (IQR) (mg/kg)	Serum concentration mean ± SD, median, (IQR) (ng/ml)	Correlation between serum concentration (ng/ml) and daily dose (mg/day) (*p*, *r*_*p*_; *r*_*s*_)	Difference in Serum concentrations (*p*)
All (93)	9.5 ± 6.0 8.8 (5.0–12.5)	0.157 ± 0.091 0.131 (0.091–0.204)	157.7 ± 112.6 126.0 (69.0–210.0)	< 0.0001; 0.809	
Girls (49)	9.4 ± 5.6 10.0 (5.0–10.0)	0.161 ± 0.095 0.141 (0.101–0.200)	158.7 ± 110.5 125.0 (72.0–210.0)	< 0.0001; 0.793	0.93
Boys (44)	9.5 ± 6.4 7.5 (5.0–13.1)	0.152 ± 0.088 0.123 (0.082–0.208)	156.5 ± 116.2 140.0 (66.8–205.5)	< 0.0001; 0.817	
Children < 14 years (24)	7.3 ± 5.2 5.0 (3.8–8.1)	0.156 ± 0.093 0.114 (0.078–0.211)	139.6 ± 101.1 106.5 (68.3–175.5)	< 0.0001; 0.879	0.40
Adolescents ≥ 14 years (69)	10.2 ± 6.0 10.0 (5.0–15.0)	0.157 ± 0.092 0.134 (0.102–0.200)	164.0 ± 116.3 157.0 (70.0–217.0)	< 0.0001; 0.775	
Monotherapy (25)	8.9 ± 6.9 5.0 (3.0–15.0)	0.141 ± 0.088 0.134 (0.074–0.183)	116.5 ± 92.7 94.0 (48.0–173.0)	< 0.0001; 0.863	0.01
Polypharmacy (all comedications) (68)	9.6 ± 5.6 8.8 (5.0–10.6)	0.162 ± 0.093 0.130 (0.099–0.221)	173.0 ± 116.0 157.5 (74.8–231.8)	< 0.0001; 0.812	
Polypharmacy with CYP-Enzyme Inhibitors (21)	8.3 ± 4.2 7.5 (5.0–10.0)	0.149 ± 0.088 0.110 (0.101–0.204)	188.7 ± 120.3 164.0 (98.0–234.0)	< 0.0001; 0.798	0.43
Polypharmacy with Non-CYP-Enzyme Inhibitors (*n* = 47)	10.2 ± 6.1 10.0 (5.0–12.5)	0.168 ± 0.095 0.141 (0.098–0.230)	165.7 ± 109.8 154.0 (74.5–228.5)	< 0.0001; 0.845	
Monotherapy and non-inhibiting comedications combined (72)	9.7 ± 6.4 10.0 (5.0–13.1)	0.159 ± 0.09 0.136 (0.083–0.203)	148.6 ± 106.2 123.0 (63.8–197.8)	< 0.0001; 0.834	0.12
Schizophrenia spectrum disorders (ICD-10 F2) (31)	13.8 ± 7.0 15.0 (10.0–20.0)	0.206 ± 0.102 0.198 (0.132–0.261)	201.2 ± 120.2 187.0 (99.0–263.5)	< 0.0001; 0.774	0.04 (ICD-10 F2/ ICD-10F9)
Affective disorders (ICD-10 F3) (26)	8.2 ± 4.1 7.5 (5.0–10.0)	0.134 ± 0.071 0.111 (0.095–0.169)	147.2 ± 105.2 125.0 (72.0–175.0)	< 0.0001; 0.820	
Personality disorders (ICD-10 F6) (11)	7.5 ± 3.2 7.5 (5.0–10.0)	0.140 ± 0.080 0.110 (0.076–0.210)	164.0 ± 170.9 66.0 (41.5–253.0)	0.01; 0.720	
Behavioural, emotional and tic disorders (ICD-10 F9) (22)	6.3 ± 3.2 5.0 (4.3–7.5)	0.129 ± 0.069 0.106 (0.075–0.177)	112.5 ± 69.2 96.5 (64.5–160.0)	< 0.0001; 0.835	

*25th -75th IQR* interquartile range, *SD* standard deviation

Significantly higher daily doses were found for patients with schizophrenia-spectrum disorders (13.8 mg) compared to the following other diagnostic groups: Affective disorders (8.2 mg; *p =* 0.007), personality disorders (ICD-10 F6) (7.5 mg; *p =* 0.04) and behavioural emotional or tic disorders (6.3 mg; *p =* 0.0003). While daily doses were significantly lower in children than adolescents (*p* = 0.04), this could not be confirmed for body weight-adjusted (in kg) doses (*p* = 0.95). Moreover, weight-adjusted doses of schizophrenia-spectrum disorders and affective disorders (0.206 vs. 0.134) (*p* = 0.01) as well as schizophrenia-spectrum disorders and behavioural, emotional or tic disorders (0.206 vs. 0.129) (*p* = 0.04) differed significantly.

The mean serum concentration of aripiprazole in all patients was 157.7 (± 112.6). For details on the serum concentrations of the subgroups (mean ± SD; median; IQR), see Table 2. The distribution of serum concentrations of the whole sample was logarithmic. Using the Wilcoxon-test, there were no differences in concentrations between subgroups classified by sex and age, however, the difference between monotherapy and higher serum concentrations in patients with polypharmacy was substantial (*p =* 0.01). Patients with CYP2D6- or CYP3A4 inhibitors had significantly higher serum concentrations than patients on monotherapy (*p* = 0.02)). Interestingly, the difference between monotherapy and higher serum concentrations in patients with non-CYP P450 enzyme-inhibiting comedications was also significant (*p* = 0.02), while there was no relevant difference between patients with inhibitors and non-inhibitors (*p =* 0.43) as comedication. The variation of serum concentrations could be explained by both the weight-adjusted doses and administration of inhibiting comedications. Combined, these two factors explained 64% (r^2 = 0.641; adjusted r^2 = 0.621; *p* < 0.0001) of the variations with the weight-adjusted doses being the regressor, adjusted for the independent variables sex and age and body weight. The percent variance explained by weight-adjusted dose alone was already around 61% (r^2 = 0.610; adjusted r^2 = 0.592), indicating that the remaining effect of inhibiting comedications was quite small.

Patients with non-inhibiting comedications received slightly, though not significantly, higher weight-adjusted doses than patients on monotherapy (*p =* 0.20) and had also numerically higher weight-adjusted doses than patients with cytochrome P450 enzyme-inhibiting comedications (*p =* 0.39) (see Table 2).

Patients with the diagnosis schizophrenia-spectrum disorder had on average higher mean aripiprazole serum concentrations (201.2 ng/ml) than patients with affective disorders (147.2 ng/ml; *p =* 0.01) and behavioural, emotional or tic disorders (112.5 ng/ml; *p =* 0.04). Patients diagnosed with schizophrenia-spectrum disorders had on average higher weight-adjusted aripiprazole doses than those with the other two main diagnostic groups, consistent with the observed differences in aripiprazole serum concentrations.

**Fig. 1:**
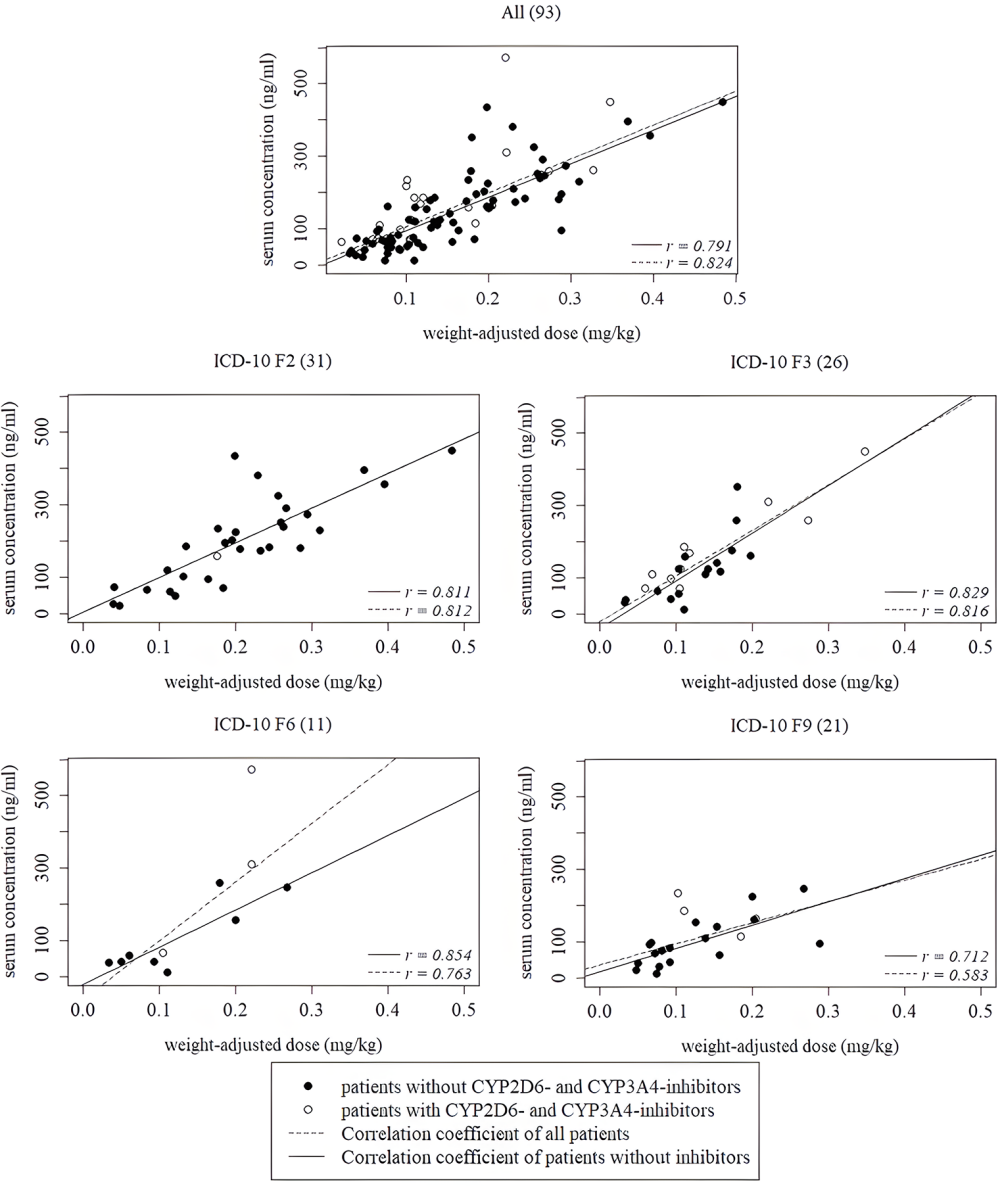
Correlation between aripiprazole serum concentrations and weight-adjusted doses of all patients and different diagnostic groups, taking in to account CYP2D6 and CYP3A4 inhibitors

A positive correlation was observed between serum concentrations and body weight-adjusted doses (*r*_*s*_ = 0.791) and daily doses (*r*_*s*_ = 0.809). All subgroups had a significant relationship between daily doses and serum concentrations as well as weight-adjusted doses and serum concentrations. Correlation coefficients for most subgroups ranged between 0.7 and 0.9 (with the exception for children without the influence of CYP-enzyme inhibitors (*n* = 20, *r* = 0.592) and patients with behavioural, emotional or tic disorders (*n* = 22, *r =* 0.58), and can therefore be summarized as an overall high correlation. See Table 2 for correlation coefficients for serum concentrations and daily doses of all analysed subgroups Fig. 1.

### Clinical effectiveness of aripiprazole

Information based on CGI-I was available for 99% (*n* = 92) of all patients. The mean (SD) CGI-I score of all patients was 2.3 (1.1). Between the four diagnostic subgroups studied, the mean (SD) CGI-I scores were very similar: Patients with schizophrenia-spectrum disorders had a mean CGI-I of 2.4 (± 1.1), affective disorders 2.5 (± 0.99) and personality disorders 2.5 (± 0.8). Patients with behavioural, emotional or tic disorders had a slightly lower (better) mean CGI-I of 2.1 (± 1.3).

Regarding the entire study sample, 62% of the patients were responders to aripiprazole (CGI-I score 1 and 2). Of these, 18% (*n* = 17) were rated as ‘very much improved’ (CGI score 1) and 44% (*n* = 41) as ‘much improved’ (CGI-score 2). Altogether, 25% of the patients were rated as ‘minimally improved’ and not considered as treatment responders (Table 1). While only 46% of patients with personality disorder diagnoses were CGI-I responders, more than three quarters (76%) of the patients with behavioural, emotional or tic disorders, close to two thirds (62%) of patients with affective disorders and some over half (58%) of patients with schizophrenia-spectrum disorders were CGI-I responders. Patients with daily doses up to 15 mg/d (*n* = 82) were rated at least “much improved” in roughly 64%, while patients with daily doses above 15 mg/d (*n* = 11) were also rated as “much improved” or better in 64%.

Girls responded to the treatment with aripiprazole about as often as boys (Chi2 *p =* 0.28). The same applied to children and adolescents (Fisher’s Test *p =* 0.50).

**Fig. 2:**
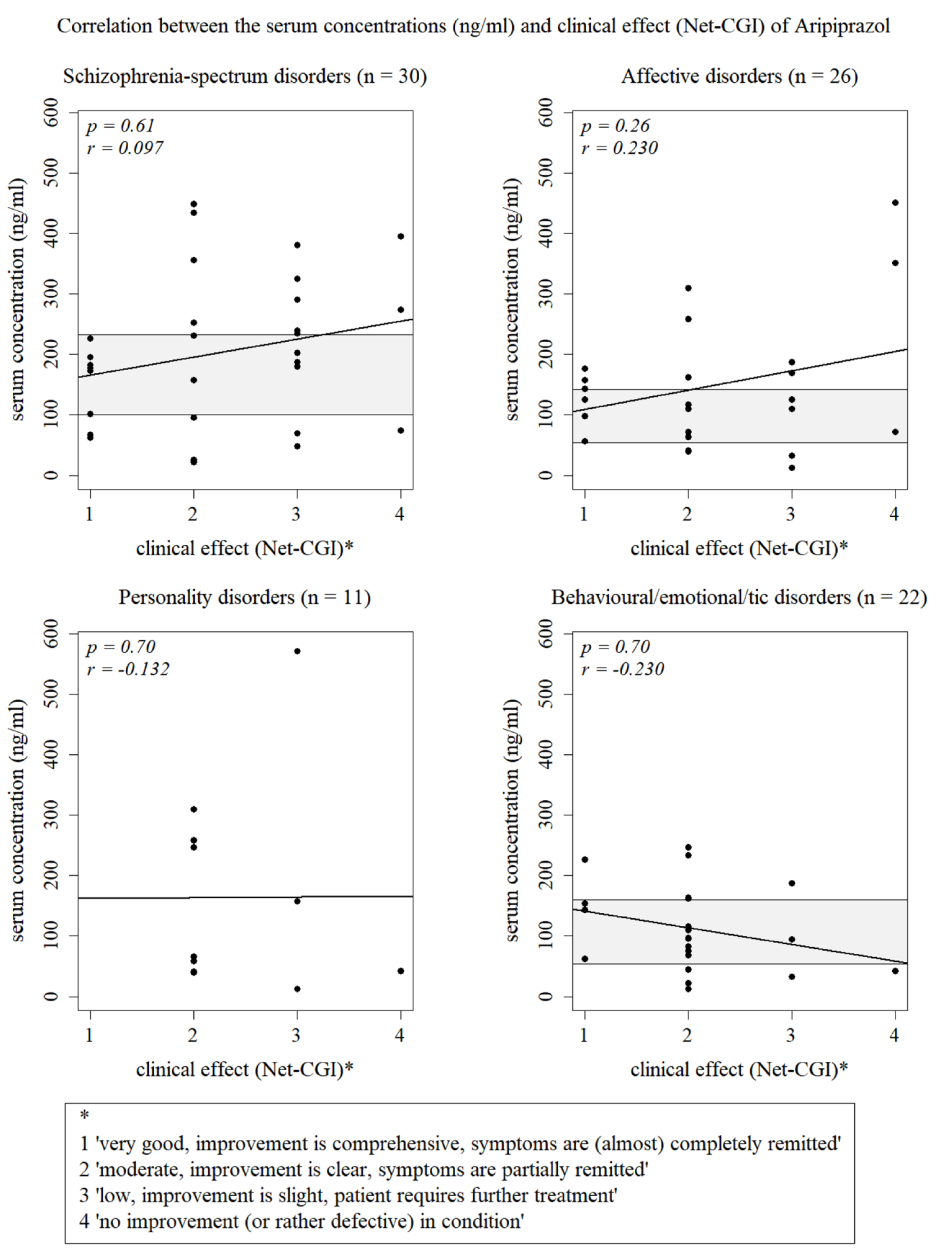
Non-significant correlation between the serum concentrations and the clinical effectiveness of aripiprazole measured by the NET-CGI for diagnosis schizophrenia-spectrum disorders (*p* = *0.61*), affective disorders (*p* = *0.26*), personality disorders (*p* = *0.70*) and behavioural/tic disorders (*p* = *0.30*)

Responding patients (CGI-I = 1–2) and non-responding patients (CGI-I = 3–7) did not have relevantly different mean (SD) serum concentrations: 142.3 ng/ml (93.4) vs. 183.7 ng/ml (138.4) or median (IQR) serum concentrations: 123.0 ng/ml, (IQR: 66.3, 189.8 ng/ml) vs. 159.0, (IQR: 72.5, 270.0 ng/ml) (Welch test: *p* = 0.12). The correlation coefficient of the correlation between serum concentrations and the CGI-I calculated by the spearman test was 0.07 and therefore rather insignificant (*p* = *0.5*). The serum concentrations and the clinical effectiveness of medication rated from 1 to 4 did also not correlate with an also rather unpleasing correlation coefficiant (*p* = *0.28*, *r* = *0.11*). The serum concentrations did not significantly differ between the four ratings (Kruskal-Wallis test: *p* = 0.74). None of four diagnostic subgroups did have a significant correlation for the two factor either: schizophrenia-spectrum disorders (*n* = 30) *p* = 0.61, *r* = *0.097*; affective disorders (*n* = 26) *p* = 0.26, *r* = *0.230*; personality disorders (*n* = 11) *p* = 0.70, *r* = -*0.132* and emotional and tic disorders (*n* = 22) *p* = *0.30*, *r* = *-0.230* (see Fig. 2).

### Adverse drug reactions of aripiprazole

At the time of blood collection analysed for this study, in 33% of the patients, one up to six ADRs were reported. Most of the total of 64 ADRs were described as mild (27%) or moderate (61%). Only 3% of all 64 reported ADRs were classified as ‘severe’ or ‘extremely severe’. The most common ADR was weight gain, which was reported by 11% of all patients, followed by exhaustion and increased appetite (both 8%), hypersomnia (6.5%) and restlessness (3%). In 2% of the patients, irritable or bad mood, sad or depressed mood, impaired emotional range, dyskinesia or dystonia, sweating and hypersalivation were observed, while one patient, respectively, reported angry or hostile mood, apathy/lack of interest, emotional lability/mood swings, anxious mood (tense), impulsiveness, attention/concentration difficulties, suicidal behaviour, pronounced thirst, tics, elevated mood, exanthema, tachycardia/arrhythmia, dizziness, headache, visual disturbances, constipation, hair loss or abnormal hair growth, eye twitching, or hand tremors.

Altogether, 42% of patients with schizophrenia-spectrum disorders had ADRs, while only 27% of patients with personality and behavioural, emotional and tic disorders reported ADRs, and 31% patients with affective disorders reported ADRs. The occurrence of ADRs among the three diagnostic groups was tested with the Kruskal-Wallis test and found not to be statistically different (*p* = 0.49). Furthermore, 29% of the girls and 39% of the boys reported ADRs (Chi2 *p =* 0.42). For children, the frequency was 21% vs. 38% for adolescents (Chi2 *p =* 0.21).

Patients without ADRs had a mean (SD) serum concentration of 162.6 ng/ml (113.6) and a median serum concentration of 125.5 ng/ml (IQR: 72.3, 223.75 ng/ml), while patients with one or more ADR had a mean (SD) serum concentration of 147.8 ng/ml (111.7) and a median serum concentration of 143.0 ng/ml (IQR: 59.0, 191.5 ng/ml). No relationship between serum concentrations and the occurrence of ADRs was found (*p =* 0.53). Further, no significant relationship between the serum concentration and the number ADRs was detected (*p =* 0.29).

### Estimation of preliminary therapeutic reference ranges of aripiprazole in children and adolescents with the diagnoses schizophrenia-spectrum, affective, and behavioural, emotional or tic disorders

Two methods to calculate preliminary therapeutic reference ranges, the mean ± SD and the median (IQR; 25th–75th interquartile range) of all responding patients (with and without inhibiting comedication), were applied in this study. Table 3 shows the resulting reference ranges of aripiprazole for the whole sample, for the three main diagnostic subgroups, and for patients with monotherapy of aripiprazole (regardless of diagnosis).

**Table 3:** Therapeutic reference ranges (TRR) of aripiprazole for the whole sample and the different subgroups calculated by mean ± SD vs. by the 25th and 75th quartile of the serum concentrations of responders (with and without inhibiting comedication)

Sample (*N* of responders/ responders without inhibiting comedication/ responders with monotherapy)	TRR of all responders calculated by mean ± SD method (mg/kg)	TRR of responders without inhibiting comedication calculated by mean ± SD method (mg/kg)	TRR of responders with Monotherapy calculated by mean ± SD method (mg/kg)	TRR of all responders calculated by IQR method (mg/kg)	TRR of responders without inhibiting comedication calculated by IQR method (mg/kg)	TRR of responders with Monotherapy calculated by IQR method (mg/kg)
All responders regardless of diagnosis (58/44/15)	49.0–235.7	40.8–243.3	37.1–165.7	66.3–186.8	63.8–189.0	46.0–165.5
Schizophrenia spectrum disorders (18/17/5)	81.0–316.8	80.2–322.4	*	116.0–229.8	102.0–231.0	*
Affective disorders (16/12/3)	60.1–178.7	54.7–186.1	*	70.0–159.0	62.3–159.0	*
Behavioural and tic disorders (16/13/4))	45.9–189.7	34.8–176.2	*	67.5–156.5	63.0–143.0	*

*Not calculated due to small size of subsample

For patients with schizophrenia-spectrum disorders, a preliminary therapeutic reference range of 102.0-231.0 ng/ml was calculated by the 25th and 27th quartile (IQR-method) for responders without cytochrome P450 enzyme inhibiting comedication. As there were more than 10 responders without inhibiting comedication for this diagnosis, the reference ranges were also calculated for patients with affective disorders (62.3–159.0 ng/ml) and behavioural, emotional or tic disorders (63.0–143.0 ng/ml). As the diagnostic group of patients with personality disorder comprised only four responders, no estimation was made for this diagnosis. The first interquartile (54.3 ng/ml) and third interquartile (250.0 ng/ml) indicate a rather small lower limit and a similar upper limit as for patients with schizophrenia-spectrum disorders.

**Fig. 3:**
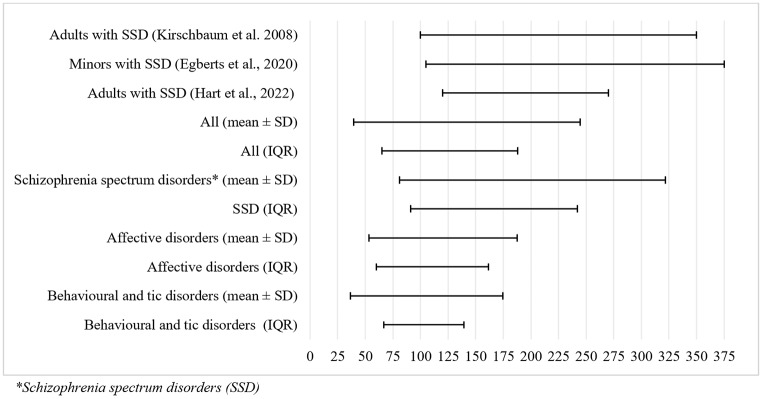
Preliminary therapeutic reference ranges calculated for responders without inhibiting comedication for aripiprazole in children and adolescents with different diagnoses in comparison to recently proposed therapeutic reference ranges for schizophrenia and the recommended therapeutic reference range for adults with schizophrenia. *Schizophrenia spectrum disorders (SSD)

The percentages of responders (CGI-I 1–2) within the two calculated reference ranges (IQR vs. mean ± SD) and the occurrences of ADRs within these reference ranges were compared between the three main diagnostic subgroups: For schizophrenia spectrum disorders, the frequency of treatment responders within the IQR range was 83% vs. 66% responders within the mean ± SD range. For tic disorders, we found 80% vs. 73% of responders, and for affective disorders, 75% vs. 82%. Concerning the occurrence of ADRs within the reference ranges (IQR vs. mean ± SD) we found 41% vs. 50% of patients with an ADR for patients with schizophrenia spectrum disorders, 25% vs. 31% for affective disorders and 20% vs. 20% for tic disorders Fig. 3.

## Discussion

This analysis of data originating from the prospective TDM-VIGIL study revealed a linear relationship between aripiprazole doses and serum concentrations in children and adolescents treated for various psychiatric diagnoses. Weight-adjusted doses and cytochrome P450 enzyme inhibitors together explained about 64% of the variations in serum concentrations, with weight-adjusted doses explaining already 61% of the variance, while sex, age and body weight had no significant influence in the multiple regression model.

As almost two thirds of all patients were rated with CGI scores as ‘very much’ and ‘much’ improved, the treatment with aripiprazole may cautiously be described to have had a positive outcome in the majority of patients in this open label study of predominantly inpatients. This evaluation is supported by the observation that discontinuation of the drug was observed rarely and only few severe ADRs occurred throughout the study.

### Serum concentrations in relation to aripiprazole doses

The wide distribution of aripiprazole concentrations in the analysed blood samples is in line with former studies in adults (Lin et al. 2011; Kirschbaum et al. 2008; Pozzi et al. 2016). Genetic polymorphisms of the cytochrome P450 3A4 and 2D6 enzymes as well as differences in medication distribution, clearance, or the body constitution have been cited as underlying factors (Sachse et al. 1997; Mizutani 2003; Hendset et al. 2014; Molden et al. 2006). On this basis, Egberts et al. (2020) argued that it may be reasonable to implement genotyping before executing future studies, to rule out the possibility of genetic variations in the metabolizing enzymes.

In line with previous studies in children and adolescents (Bachmann et al. 2008; Egberts et al. 2020; Findling et al. 2008; Pozzi et al. 2016) as well as in adults (Kirschbaum et al. 2008; Lin et al. 2011), in this study a strong correlation between aripiprazole serum concentrations and daily doses, as well as a moderate correlation weight-corrected doses, was found for all subgroups. While variability in serum concentrations could be attributed to variation in dose and the administration of cytochrome P450 enzyme-inhibiting concomitant medications, other variables, such as age, sex and or body weight, had no influence on serum concentrations. These findings were similar to the findings of Bachmann et al. (2008), who also found no differences in aripiprazole serum levels between girls and boys, nor weight or BMI, nor children and adolescents. Their sample had a mean age of 18.7 years with a range of 13.5–21.6 years. The authors stated, that at the age of 18, the aripiprazole-metabolizing enzymes have reached the activity of adults (Bachmann et al. 2008; Strolin Benedetti et al. 2005). Egberts et al. (2020), however, observed significantly higher serum concentrations in children than in adolescents, although body weight-adjusted doses were similar. In our study, the difference of 50.5 ng/ml between the median serum concentration of children (*n* = 24) and adolescents (*n* = 69) was not statistically significantly different (*p* = 0.40). However, it is possible, that the utilized Wilcoxon-Test was distorted by the small sample of children in our study, so that we are unable to make any conclusive statement on possible differences in aripiprazole serum levels between children and adolescents.

### Comedication

About 73% of the patients in this study received concomitant medications. In line with former studies (Nemoto et al. 2012; Pozzi et al. 2016; Kirschbaum et al. 2008), patients with concomitant CYP450 2D6- or CYP450 3A4 inhibitors (22%) had significantly higher serum concentrations than patients with aripiprazole monotherapy, although the weight-adjusted doses were similar in both groups (0.149 mg/kg vs. 0.141 mg/kg). However, significantly higher aripiprazole serum concentrations were also found in patients on polypharmacy with non-inhibiting comedications. This finding may be explained by the correlation between weight-adjusted doses and serum concentration: Patients with non-inhibiting comedications received slightly higher weight-adjusted aripiprazole doses than patients with CYP450 inhibiting comedications and monotherapy (0.168 mg/kg vs. 0.149 mg/kg and 0.141 mg/kg). As patients with CYP450 inhibiting comedication had numerically higher serum aripiprazole concentrations than patients without (188.7 ng/ml vs. 165.7 ng/ml), it is likely that the difference in serum concentrations between patients with monotherapy and non-inhibiting comedications resulted from higher weight-adjusted aripiprazole doses rather than the comedication itself.

### Clinical effects

Symptom trajectories of the overall sample were rated as rather favourable. Our finding, that 55% of the patients with schizophrenia-spectrum disorder were rated as at least “much improved” is equal to the result of Egberts et al. (2020), who also reported that in their sample approximately 55% of the paediatric patients diagnosed with schizophrenia-spectrum disorders were classified as ‘very much’ or ‘much improved’. Patients diagnosed with behavioural, emotional or tic disorders were rated at least ‘much better’ in 59% in our study vs. 41.6% in Egberts et al.’s (2020) sample, though their sample consisted of slightly more patients with tic disorders than ours (2% vs. 5%). The lower percentage of responders in patients with schizophrenia-spectrum disorders compared to the other main diagnostic groups could potentially be due to pharmacodynamic non-responders, who have been described as very common with this diagnosis (Shaw et al. 2006). The similar frequency of treatment response in patients with aripiprazole doses below and above 15 mg/d may be a result of placebo-responders or patients who benefited from the therapeutic setting or improved spontaneously. Such patients cannot be differentiated from the patients who actually responded to the low doses. However, it is known that patients who are less responsive to treatment receive higher doses, which also tends to obscure potential dose-response relationships in studies utilizing flexible doses (Hiemke 2019). Together, these factors are likely responsible for the missing significant correlation between aripiprazole serum concentrations and clinical effectiveness. The missing correlation is illustrated by the graphs in Fig. 2. If the therapeutic reference ranges, we calculated for the three main diagnosis in our study, are taken into account, it may be noticed, that majority of patients with very good to low improvement were within or close to these reference ranges while almost none of those having shown no improvement were within these ranges. This could cautiously be interpreted as further evidence of the validity of those reference ranges. Also, the better the improvement of the patient was rated, the less was the distribution of the serum concentrations within this rating while the mean and median weight adjusted daily doses varied only little throughout the four ratings.

In line with the often reported relatively small risk of aripiprazole for ADRs, only one third of the patients reported ADRs, with a majority of these being rated as ‘mild’ or ‘moderate’. Only 3.1% of all 64 reported ADRs were rated ‘severe’ and ‘extremely severe’. These findings were very similar to the study of Egberts et al. (2020), who also found only 3.4% of ‘severe’ ADRs.

### Comparison of the two calculation methods for therapeutic reference ranges

The method to estimate preliminary therapeutic reference ranges proposed by the AGNP expert group, using the arithmetic mean ± one standard deviation of treatment responders, requires that serum concentrations follow a Gaussian distribution. Aripiprazole serum concentrations have been shown to often follow a logarithmical distribution (Hart et al. 2020) and it is therefore proposed to calculate the 25th and 75th quartiles as the lower and the upper limit of the therapeutic reference range, as carried out in a recently published review (Hart et al., 2022). The distribution of serum concentrations of the whole sample in our study was in fact also logarithmic, yet the distribution in patients with schizophrenia-spectrum disorders, personality and behavioural, emotional or tic disorders followed a Gaussian distribution, while the distribution in patients with affective disorders was logarithmic. For this reason, as well as for better comparability with prior TDM studies, we calculated the preliminary therapeutic reference ranges with both, statistically only partially applicable methods. Nevertheless, in all three diagnostic subgroups, we observed that ADRs occurred less often within the interquartile-derived reference range than within the mean ± SD-derived reference range. Also, although the mean ± SD reference range was wider than the interquartile reference range, the percentage of responders within the interquartile range was higher in most groups than in the mean ± SD reference range. These data indicate superiority of the median/IQR approach, but this finding should be replicated by always comparing the two methods in further studies (although a reanalysis in studies reporting only one metric could also be informative).

### Proposal of a preliminarily therapeutic ranges for children and adolescents

Based on the mean aripiprazole serum concentration in the treatment responders of their naturalistic sample, Egberts et al. (2020) calculated a preliminary therapeutic reference range for children and adolescents with schizophrenia-spectrum disorders of 105–375 ng/ml. As this range is similar to the valid therapeutic reference range 100–350 ng/ml for adult patients with schizophrenia-spectrum disorders (Kirschbaum et al. 2008), they concluded, this range could also be valid for paediatric patients. However, the authors used the mean ± SD of patients with CGI-I scores of 1, 2 and 3 for calculation, which probably lead to a higher mean aripiprazole serum concentration, as even patients minimally responding where included in whom like doses were pushed up, without getting an appropriate clinical response. The preliminary therapeutic reference range for patients diagnosed with schizophrenia-spectrum disorders in our study was 102–231 ng/ml, which has a perceptibly lower upper value than in the study by Egberts et al. (2020). Yet it is very similar to two recently proposed reference ranges for adults in the systematic review of Hart et al. (2021) (120–270 ng/ml) and Lin et al. (2011) (134–271 ng/ml) who examined plasma levels and the clinical response of aripiprazole in oriental, adult patients. Another similar result was found by Kirschbaum and colleagues (Kirschbaum et al. 2008) in their study of adult patients, as they reported no or only mild ADRs within a concentration range of aripiprazole between 110 and 249 ng/ml (25th to 75th percentile).

In our study, both patients with non-CYP450-inhibiting comedications and patients with inhibiting comedications were found to have significantly higher serum concentrations at similar weight-adjusted aripiprazole doses than patients on monotherapy. However, to exclude all patients with any comedication was not possible due to our small diagnostic sub-samples. The reference ranges for the diagnostic subgroups were based on less than 20 patients per subgroup: 17 for schizophrenia-spectrum disorders, 13 for affective disorders and 12 patients for behavioral and tic disorders. Therefore, we only were able to calculate a reference range for patients with treatment response on aripiprazole monotherapy for the whole sample.

For the subgroups, the reference ranges calculated for all treatment responders, regardless of their comedication, tended to have a higher lower threshold than those of patients without CYP450-inhibitory medication. Due to the lack of comparative data in the literature and the small sample sizes, future studies with larger samples are needed to confirm these preliminary therapeutic reference ranges for aripiprazole in children and adolescents with psychiatric disorders.

## Study limitations

Several limitations of this study need to be recognised. First, the sample size of several subgroups was limited, for example in children. With these small samples, misinterpretations could have occurred due to both type I and type II errors, as some tests are sensitive to small samples, such as the Kruskal-Wallis test and the Chi2 test. In order to avoid errors, additional tests were carried out, such as Fisher’s exact test.

Second, there are possible methodology deficiencies. An example is the selection of only the most recent blood sample of each patient with more than one aripiprazole measurement. This procedure may have resulted in the pooling of patients whose psychopathology already had improved over time and who may have developed tolerance to early side effects. The actual share of ADRs and outcomes throughout the entire TDM-VIGIL study may therefore differ significantly from the results of this study.

Third, the flexible dose design is suspected to affect the correlation between serum concentrations and clinical effects (Hiemke 2019; Hart et al. 2021) and reduces the validity of such studies in terms of the validity of the relationship between serum concentrations and clinical response. Fixed dose studies would be more informative but are difficult to conduct in clinically generalizable samples.

Fourth, only few (*n* = 5) patients were included in more than one main diagnostic subgroup because of comorbidities. Larger study populations are needed to secure disjoint subgroups in order to establish reliable data that may not be affected by being analysed in more than one diagnostic subgroup.

Finally, the metabolite dehydro-aripiprazole was not measured in the study. Therefore, therapeutic reference ranges are explicitly provided for aripiprazole only, which contradicts the current TDM-guidelines (Hiemke et al. 2018), which emphasise the importance of the active metabolite and its contributions to the effectiveness of the parent drug.

## Conclusion

This study confirmed a significant correlation between (body weight-adjusted) daily doses and serum concentrations of aripiprazole in children and adolescents, without significant differences based on sex, age or BMI. Moreover, patients with CYP450 2D6- and CYP450 3A4-inhibiting comedications had significantly higher aripiprazole serum concentrations at the same weight-normalized daily doses. Based on the 25th and 75th quartile of serum concentrations in clinical treatment responders, a preliminary therapeutic reference range for patients with schizophrenia-spectrum disorders (∼ 100–230 ng/ml) was suggested, whose lower threshold is identical to the one in adults, but whose upper threshold is slightly lower compared to recently published data for adults. For the other main diagnostic groups, aripiprazole reference ranges were found to be lower, but these findings were hampered by low sample sizes. Further studies need to confirm all age-specific reference serum level ranges for aripiprazole, especially the levels estimated in this study for patients with affective disorders (∼ 60–160 ng/ml) and behavioural, emotional or tic disorders (∼ 60–140 ng/ml).

## Data Availability

The data that support the findings of this study are not openly available due to reasons of sensitivity and are available from the corresponding author upon reasonable request. Data are located in controlled access data storage at Department of Child and Adolescent Psychiatry and Psychotherapy, University Medical Center Freiburg, Freiburg, Germany.
